# Global Trends in Immunotherapy Research on Breast Cancer over the Past 10 Years

**DOI:** 10.1155/2020/4708394

**Published:** 2020-11-05

**Authors:** Soo Kyung Ahn, Ji Woong Hwang

**Affiliations:** Department of Surgery, Kangnam Sacred Heart Hospital, Hallym University College of Medicine, Seoul 07441, Republic of Korea

## Abstract

In recent years, many studies have focused on the host immune system and its relationship with tumor progression in a variety of solid tumors, including breast cancer. This study investigates recent trends of immunotherapy research in breast cancer and compares the contributions of research from different regions, institutions, and authors. A search of breast cancer and immunotherapy studies that were published between 2010 and 2019—with different keyword combinations—was performed in the Web of Science database. Bibliometric data were collected for analysis. VOSviewer software was used to generate a figure for the keyword's co-occurrence network, so as to implement network visualization analysis. A total of 1,041 publications were identified. The United States and China contributed to approximately 50% of the publications, 336 and 208, respectively. Both countries drove the increase in publications after 2015. A paper entitled “Pembrolizumab in patients with advanced triple-negative breast cancer: Phase IB KEYNOTE-012 Study” that was published in the *Journal of Clinical Oncology* by Nanda et al. was the most cited (715 citations). The keywords found in this research were grouped into four clusters: “mechanism,” “vaccination,” “PD-L1,” and “chemotherapy.” The terms “tumor-infiltrating lymphocytes” and “PD-1/PD-L1” are among the latest hotspots, which mostly appeared in 2017. Author keyword analysis revealed that recent trends in breast cancer immunotherapy focus on the triple-negative breast cancer subtype and PD-1/PD-L1 immune checkpoint pathway and inhibitors. This study analyzed global trends in immunotherapy research on breast cancer over the past 10 years and provided insight into the features and research hotspots of the articles in this issue.

## 1. Introduction

Breast cancer is the most commonly diagnosed cancer and the leading cause of mortality in females worldwide [1]. Nearly 20% of patients who develop breast cancer die despite recent progress in scientific research. During the last decades, improved understanding of mammary oncogenesis and metastatic progression has led to major therapeutic progress, such as hormone therapy, which targets the estrogen receptor (ER) and targeted therapies directed against oncogenic proteins (HER2, EGFR, CDK4/6, and PI3K/AKT/mTOR pathway). However, due to the high mutagenic and adaptable ability of cancer cells, resistant clones emerge in most cases.

Many studies have focused on the presence and function of the host immune system and its relationship with tumor progression in recent years. Breast cancer has long been recognized as less immunogenic than melanoma or renal cell carcinoma, and the results of adoptive immunotherapy (interleukin 2 and interferons) were relatively disappointing. The role of immunity in breast cancer progression has emerged during the last decades with the demonstration of an impact of the tumor microenvironment on survival and/or therapeutic response [2].

Bibliometric analysis has employed citation count as a proxy measure of research quality [3, 4]. The study of the quantitative aspects of the production and dissemination of the knowledge generated in the time interval observed through bibliometric analysis has allowed tracing of the research profiles of different countries, centers, and researchers offering important analysis of scientific production, behavior, and development in research fields [5, 6].

The purpose of this study is to use bibliometric methods to analyze recent 10-year trends in immunotherapy research in breast cancer and consequently create a better understanding of the current situation and trends of those research studies by analyzing their main characteristics.

## 2. Materials and Methods

**Figure 1:**
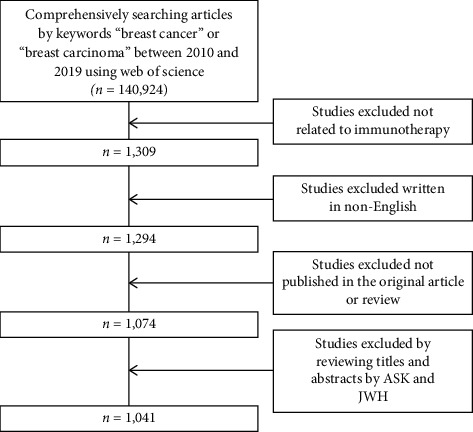
Flowchart showing the progress of article selection.

Bibliometric analysis was performed based on the Science Citation Index-Expanded (SCI-E) of the Web of Science (WoS), which is considered as the optimal database for bibliometrics [7]. All articles between 2010 and 2019 were searched in WoS from database inception to March 19, 2020, using the keywords “breast cancer” or “breast carcinoma” and immunotherapy (Figure 1). Only articles written in English were included in this study. Publications, except original articles and reviews, were excluded. Two authors, SKA and JWH, independently screened and extracted data entry and collection. Any disagreement was resolved by discussion until a consensus was achieved. Data were imported to Microsoft Excel Office 365 for analysis.

The full records of each publication, such as title, year of publication, author's name, institution, country of origin, publishing journal, keywords, and abstract, were analyzed. All articles were analyzed according to their topic: immune mechanism, immune therapy, vaccination/virus, and others (including review). VOSviewer version 1.6.14 software (Leiden University, Leiden, Netherlands) was used to analyze the relationship between the keywords to generate the map and cluster visualization. Each circle and label represented a keyword, and the size of circles represented the frequency of occurrence. The larger the circle was, more frequently the circle-represented body appeared. Circles adopting different colors in the graph represented different clusters. Average year map of keywords is also generated.

## 3. Results

**Figure 2:**
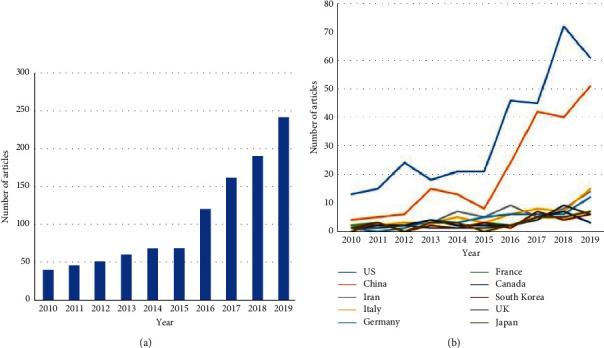
(a) The annual number of publications for immunotherapy on breast cancer from 2010 to 2019. (b) The annual trend publications related to immunotherapy on breast cancer by country from 2010 to 2019.

This study included 1,041 publications in immunotherapy on breast cancer.  Table 1 lists the top 10 cited articles in immunotherapy on breast cancer from 2010 to 2019. These articles had 2,744 citations, representing 14.9% of total citations. A paper entitled “Pembrolizumab in patients with advanced triple-negative breast cancer: Phase IB KEYNOTE-012 Study” [8] published in the *Journal of Clinical Oncology* by Nanda et al. was the most cited (715 citations). A significant increase in the number of annual publications was globally observed after 2015 (Figure 2(a)).  Figure 2(b) shows that the United States and China were driving this increase.

The University of Texas MD Anderson Cancer Center produced 21 papers with 564 citations followed by the National Cancer Institute (*n* = 13,339 citations) and Johns Hopkins University (*n* = 12,348 citations) (Table 2). Approximately 850 authors contributed to the total number of publications.  Table 3 lists the active corresponding authors who published more than five articles. Most of the authors are from the United States. Interestingly, Curigliano, G—who published the most papers (*n* = 10, citations = 181)—is from Italy.

A total of 364 journals about immunotherapy in breast cancer were published. The largest number of papers was published in *Oncoimmunology* (*n* = 36,525 citations) (Table 4). *Breast Cancer Research and Treatment* had the most citations (*n* = 1,175).

**Figure 3:**
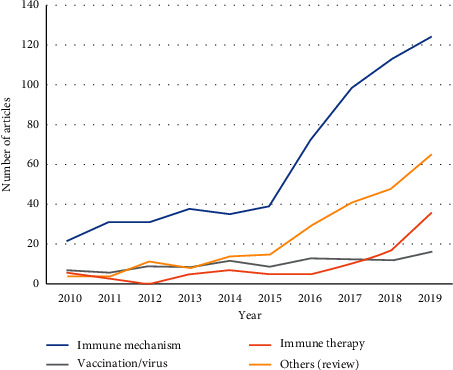
The annual trend publications related to immunotherapy on breast cancer by main topic from 2010 to 2019.

The articles were divided into the following categories according to the main topic (Figure 3): immune mechanism (*n* = 603), immune therapy (*n* = 94), vaccination/virus (*n* = 105), and others (*n* = 239). The articles related to immune mechanism, immune therapy, and others (including review) increased after 2015. In contrast, the number of publications on vaccination and virus remained unchanged.

### 3.1. Hotspots

Keywords of 1,041 articles were analyzed using a co-occurrence network analysis tool in VOSviewer. Co-occurrence analysis helps to find direction and popular topics in research and has proven to be important in monitoring scientific development [9]. Of 4,454 keywords, 100 were used ≥ 20 times in titles and abstracts of all articles. Keywords were classified into four clusters formed in software VOSviewer: “mechanism,” “vaccination,” “PD-L1,” and “chemotherapy.”

**Figure 4:**
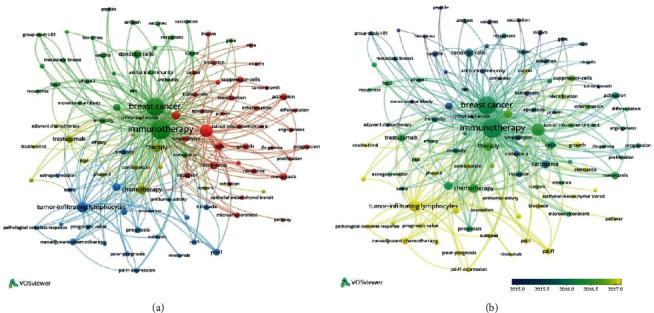
Co-occurrence analysis of global research about immunotherapy on breast cancer. (a) Mapping of keywords in the research. (b) Average year map of keywords.

In the cluster “mechanism,” the most used keywords were “expression” (239 times), “t-cells” (101 times), and “cells” (95 times). In the cluster “vaccination,” the most used keywords were “immunotherapy” (600 times), “breast cancer” (493 times), and “dendritic cells” (102 times). In the cluster “Pd-L1”, the most used keywords were “tumor-infiltrating lymphocytes” (170 times), “survival” (98 times), and “neoadjuvant chemotherapy” (65 times). In the cluster “chemotherapy,” the most used keywords were “chemotherapy” (139 times), “therapy” (129 times), and “trastuzumab” (93 times). Keywords and association lines are shown in Figure 4(a) and listed in Table S1. VOSviewer applied colors to keywords based on the year that they appeared in the literature (Figure 4(b)). Keywords in blue appeared early followed by green and yellow colors, which appeared later. The terms “tumor-infiltrating lymphocytes” and “PD-1/PD-L1” are among the latest hotspots, which mostly appeared in 2017. The cluster “mechanism” had the more recently used keyword “pathway” (23 times cited, appeared in 2017), the cluster “vaccination” had “recurrence” (23 times cited, appeared in 2016), the cluster “PD-L1” had “pembrolizumab” (28 times cited, appeared in 2018), and the cluster “chemotherapy” had “nanoparticles” (21 times cited, appeared in 2017).

**Figure 5:**
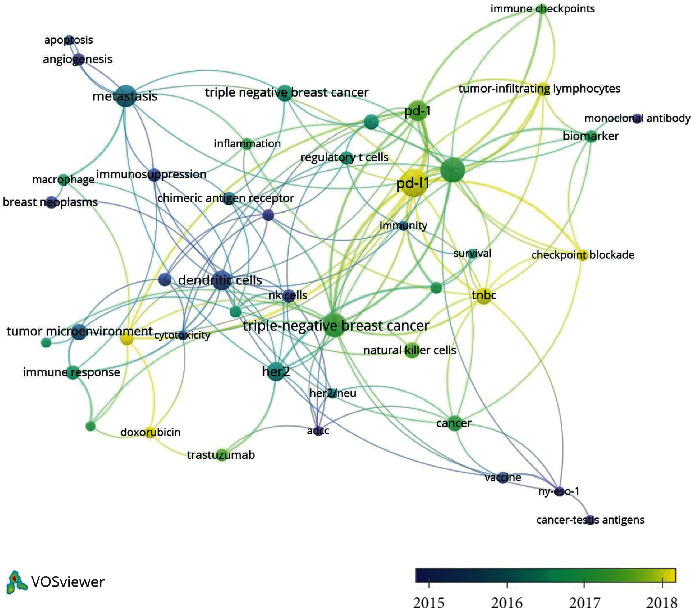
Average year map of keywords by co-occurrence analysis of global research about topic 1 (“immune mechanism”) among immunotherapy on breast cancer.

We performed additional keyword analysis on topics 1 (immune mechanism) and 2 (immune therapy) for further analysis. In this analysis, author keywords were used for analysis in VOSviewer. By supplying details of the article's subject, author keywords offered information about the research trend, which most interested researchers [10]. Articles classified into topic 1 (immune mechanism) were analyzed in VOSviewer. Of 1,340 keywords, 48 were used ≥5 times in author keywords (Figure 5, Table S2). Since we intended to identify the content of the research, the main title keywords “breast cancer” and “immunotherapy” were excluded.

In the early phase, the keywords “dendritic cells” (18 times) and “NK cells” (8 times) appeared. The latest trends showed that “PD-L1” (36 times) would be investigated in the future. The keyword “triple negative breast cancer” or “triple-negative breast cancer” appeared the most (39 times).

**Figure 6:**
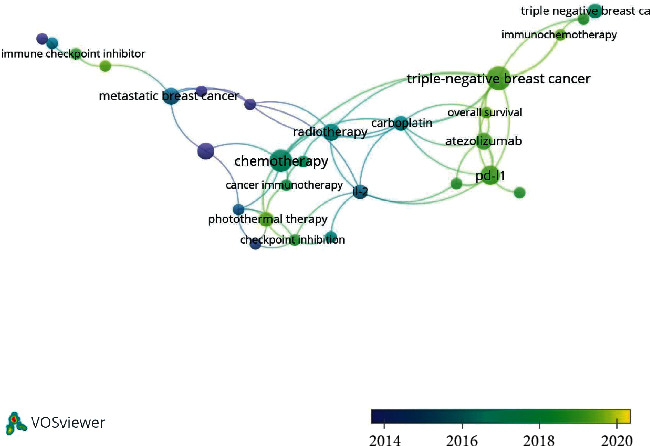
Average year map of keywords by co-occurrence analysis of global research about topic 2 (“immune therapy”) among immunotherapy on breast cancer.

In the analysis of the articles in topic 2 (immune therapy), of 255 keywords, 38 were used ≥ 2 times in author keywords (Figure 6, Table S3). The keywords “breast cancer” and “immunotherapy” were excluded. “Muc1” and “trastuzumab” appeared in the early stage of research, two and four times, respectively. The latest trends showed that PD-L1 (five times) would be investigated in the future. The keyword “triple negative breast cancer” or “triple-negative breast cancer” also appeared the most (13 times).

## 4. Discussion

In this study, we identified and analyzed the most recent 10-year trend of immunotherapy for breast cancer.

Bibliometric analysis was used to explore the status and characteristics of publication in this field, including the most cited articles, research institutions, and authors. The 10-year trend in this field has been clarified, providing ideas and direction for researchers.

After 2015, a significant increase in the number of annual publications was globally observed. Articles related to immune mechanism, immune therapy, and others including reviews increased after 2015. In contrast, the number of publication of studies on vaccination and virus remained unchanged (Figure 2).

This analysis has demonstrated that the United States and China play leading roles in breast cancer immunotherapy research. The United States (*n* = 336) and China (*n* = 208) contributed to approximately 50% of publications. Publications from other countries, such as Iran, Italy, Germany, and South Korea, are increasing gradually; however, the increase is not explosive. According to other bibliometric analyses about cancer immunotherapy, the United States contributed most of the articles with 83 papers among the 100 cited articles that were published between 1986 and 2016. [11]. However, China has occupied second position regarding the number of publications on breast cancer immunotherapy in the last 10 years (Figure 1). It is expected that China and other countries will contribute to the top 100 cited articles on breast cancer immunotherapy.

The University of Texas MD Anderson Cancer Center in the United States had the largest number of publications and citations. Institutes from China and Iran are included in the top 10 of the most cited institutes. However, their average citation is lower than that of the United States.

Keywords summarize the major content, academic thoughts, and the principal research methods of researchers. These items are the core and essence of the literature. Using VOSviewer, four clusters of keywords were classified: “mechanism,” “vaccination,” “Pd-L1,” and “chemotherapy.”

The terms “tumor-infiltrating lymphocytes” and “PD-1/PD-L1” are among the latest hotspots, which mostly appeared in 2017. The cluster “PD-L1” had more recently used keywords, such as “pembrolizumab,” and the cluster “chemotherapy” had “nanoparticles” as the more recently used keyword. In the constantly evolving era of immunotherapy, blockade of the programmed cell death 1 (PD-1)/programmed cell death ligand-1 (PD-L1) immune checkpoint pathway represents one of the most promising strategies regarding reverting immune evasion in the cancer immunoediting process. It is generally associated with the presence of tumor-infiltrating lymphocytes and poor prognostic features, such as high grade and aggressive molecular subtypes (triple-negative, basal, and human epidermal growth receptor 2- (HER2-) enriched). In the last decades, immune checkpoint inhibitors have emerged as promising treatment strategies for metastatic breast cancer, including the triple-negative subtype [12]. PD-1/PD-L1 inhibitors, including pembrolizumab, showed promising activity in the first clinical trials in breast cancer. Many trials test their efficacy and toxicity in metastatic and neoadjuvant settings as monotherapy or in combination with chemotherapy, targeted therapy (e.g., trastuzumab), radiotherapy, or nanoparticles. Nanoparticles may serve as carriers of compounds with higher selectivity for primary tumors and metastases, reducing drug resistance and side effects [13].

The usual method of using VOSviewer for analyzing keywords from the title and abstracts may include unrelated words. To understand the current knowledge structure and hot topics in the field of immunotherapy in breast cancer, we extracted and calculated “author keywords.” In both topics 1 (immune mechanism) and 2 (immune therapy), “triple-negative breast cancer” appeared the most. “PD-L1” appeared as the more recently used keyword. This shows that recent trends on breast cancer immunotherapy are focusing on the triple-negative breast cancer subtype and PD-1/PD-L1 immune checkpoint pathway and inhibitors.

As with other bibliometric analyses, our study also has some potential limitations. First, citation analysis was based on Web of Science, and we may have missed some important papers which have been indexed by other databases, such as Scopus and Google [14]. Second, the inherent problems associated with citation analyses, such as the bias linked to rely on the total number of times an article is cited, must be noted as well. Third, searching based on title and abstracts means a small number of manuscripts which involve breast cancer immunotherapy might not have been identified [15]. Furthermore, recent publications of 2020 are not included in the manuscript.

## 5. Conclusion

In conclusion, this study analyzed the global scientific publication from the period ranging from 2010 to 2019 related to immunotherapy and applied to breast cancer research, quantitatively and qualitatively. Results showed an increase in the cumulative volume of papers worldwide and a tendency toward continued growth in terms of average publication numbers. Results also show that recent trends in breast cancer immunotherapy focus on the triple-negative breast cancer subtype and PD-1/PD-L1 immune checkpoint pathway and inhibitors.

## Figures and Tables

**Table 1:** Top 10 cited articles in immunotherapy on breast cancer from 2010 to 2019.

Author	Country	Title	Total citations
Nanda, R.	USA	[8] in patients with advanced triple-negative breast cancer: Phase IB KEYNOTE-012 Study	715
Gianni, L.	Italy	Triple-negative breast cancer: challenges and opportunities of a heterogeneous disease	452
Muenst, S.	Switzerland	Expression of programmed death ligand 1 (PD-L1) is associated with poor prognosis in human breast cancer	263
Ren, X. B.	China	Myeloid-derived suppressor cells suppress antitumor immune responses through IDO expression and correlate with lymph node metastasis in patients with breast cancer	238
Miller, J. S.	USA	A phase II study of allogeneic natural killer cell therapy to treat patients with recurrent ovarian and breast cancer	219
Stagg, J.	Canada	CD73 promotes anthracycline resistance and poor prognosis in triple-negative breast cancer	191
Maher, J.	England	Dual targeting of ErbB2 and MUC1 in breast cancer using chimeric antigen receptors engineered to provide complementary signaling	190
Zardavas, D.	Belgium	Clinical management of breast cancer heterogeneity	160
Dieli, F.	Italy	In vivo manipulation of V gamma 9 V delta 2 T cells with zoledronate and low-dose interleukin-2 for immunotherapy of advanced breast cancer patients	159
Rimm, D. L.	USA	PD-L1 expression correlates with tumor-infiltrating lymphocytes and response to neoadjuvant chemotherapy in breast cancer	157

**Table 2:** Top 10 institutions publishing in immunotherapy on breast cancer from 2010 to 2019.

Rank	Institution	Country	Number of studies	Total citations	Average citation
1	University of Texas MD Anderson Cancer Center	USA	21	564	26.9
2	National Cancer Institute	USA	13	339	26.1
3	Johns Hopkins University	USA	12	348	29
4	H. Lee Moffitt Cancer Center & Research Institute	USA	10	105	10.5
5	Sichuan University	China	10	100	10
6	Shiraz University of Medical Sciences	Iran	10	83	8.3
7	Jilin University	China	10	41	4.1
8	Yale University	USA	9	429	47.7
9	New York University	USA	9	239	26.6
10	China Medical University	China	9	25	2.8

**Table 3:** Corresponding authors publishing more than five articles in research for immunotherapy on breast cancer from 2010 to 2019.

Rank	Corresponding author	Country	No.	Total citations
1	Curigliano, G.	Italy	10	181
2	Loi, S.	Australia	8	266
3	Mittendorf, E. A.	USA	8	124
4	Adams, S.	USA	7	142
5	Czerniecki, B. J.	USA	7	104
6	Peoples, G. E.	USA	6	292
7	Emens, L. A.	USA	6	206
8	Hodge, J. W.	USA	5	109

**Table 4:** Journals publishing 15 more articles for immunotherapy on breast cancer from 2010 to 2019.

Rank	Journal	Impact factor 2018	Number	Total citations	Average citation
1	*Oncoimmunology*	5.333	36	525	14.6
2	*Breast Cancer Research and Treatment*	3.471	34	1,175	34.6
3	*Oncotarget* ^†^	5.168	32	565	17.7
4	*PLOS ONE*	2.776	26	557	21.4
5	*Cancer Immunology, Immunotherapy*	4.900	25	564	22.6
6	*Breast Cancer Research*	5.676	22	360	16.4
7	*Clinical Cancer Research*	8.911	19	755	39.7
8	*Frontiers in Immunology*	4.716	16	110	6.9
9	*Oncology Letters*	1.871	15	68	4.5

^†^The impact factor of *Oncotarget* was from data of 2016 because it was deselected from the Science Citation Index-Expanded in 2018.

## Data Availability

All data generated or analyzed during this study are included in this published article.

## Supplementary Materials

Supplementary Materials Supplementary table 1 (Table S1): list of keywords of all articles generated by VOSviewer. Supplementary table 2 (Table S2): list of author keywords in the articles of topic 1 generated by VOSviewer. Supplementary table 3 (Table S3): list of author keywords in the articles of topic 2 generated by VOSviewer.
