# Supplementary material for: Global Trends in Immunotherapy Research on Breast Cancer over the Past 10 Years
Source: J Oncol. 2020 Nov 5;2020:4708394. doi: 10.1155/2020/4708394 (PMC7661143; doi:10.1155/2020/4708394)
Supplement: Supplementary Materials — Supplementary table 1 (Table S1): list of keywords of all articles generated by VOSviewer. Supplementary table 2 (Table S2): list of author keywords in the articles of topic 1 generated by VOSviewer. Supplementary table 3 (Table S3): list of author keywords in the articles of topic 2 generated by VOSviewer. [file 4708394.f1.docx]

Table S1. List of keywords of all articles generated by VOSviewer.

| ID | Cluster | Links | Total Link strength | Occurrences | Avg. pub. Year |
| --- | --- | --- | --- | --- | --- |
| expression | 1 | 96 | 1159 | 239 | 2016.3739 |
| t-cells | 1 | 94 | 516 | 101 | 2016.5941 |
| cells | 1 | 83 | 423 | 95 | 2016.0737 |
| carcinoma | 1 | 89 | 415 | 87 | 2015.5057 |
| metastasis | 1 | 82 | 420 | 80 | 2016.3418 |
| activation | 1 | 81 | 345 | 74 | 2015.8514 |
| suppressor-cells | 1 | 78 | 309 | 61 | 2016.6066 |
| receptor | 1 | 82 | 312 | 59 | 2015.7119 |
| growth | 1 | 77 | 270 | 55 | 2016.9818 |
| lymphocytes | 1 | 82 | 286 | 54 | 2015.4074 |
| identification | 1 | 66 | 193 | 45 | 2016.7556 |
| microenvironment | 1 | 75 | 233 | 43 | 2016.381 |
| progression | 1 | 57 | 184 | 40 | 2015.75 |
| resistance | 1 | 72 | 212 | 40 | 2017.125 |
| in-vivo | 1 | 65 | 178 | 39 | 2014.8462 |
| tumor-cells | 1 | 64 | 165 | 38 | 2014.6579 |
| inflammation | 1 | 58 | 175 | 38 | 2016.5526 |
| in-vitro | 1 | 70 | 172 | 36 | 2015.9167 |
| induction | 1 | 66 | 175 | 35 | 2015.5714 |
| apoptosis | 1 | 61 | 168 | 34 | 2015.5588 |
| tumor microenvironment | 1 | 63 | 156 | 33 | 2016.5 |
| cancer | 1 | 63 | 141 | 31 | 2016.9677 |
| protein | 1 | 63 | 127 | 30 | 2015.4 |
| gene-expression | 1 | 50 | 124 | 30 | 2016.0667 |
| mice | 1 | 53 | 137 | 28 | 2015.4643 |
| angiogenesis | 1 | 49 | 137 | 27 | 2015.6667 |
| inhibition | 1 | 66 | 146 | 27 | 2016.2593 |
| epithelial-mesenchymal transition | 1 | 46 | 111 | 26 | 2017.3462 |
| nk cells | 1 | 40 | 113 | 25 | 2015.76 |
| differentiation | 1 | 53 | 111 | 25 | 2016.28 |
| stem-cells | 1 | 52 | 117 | 25 | 2016.32 |
| tumor-growth | 1 | 49 | 129 | 24 | 2015.0833 |
| ifn-gamma | 1 | 52 | 113 | 23 | 2015.9565 |
| proliferation | 1 | 46 | 111 | 23 | 2016.0435 |
| pathway | 1 | 38 | 112 | 23 | 2017.3913 |
| immunotherapy | 2 | 99 | 2850 | 600 | 2016.1374 |
| breast cancer | 2 | 99 | 2375 | 493 | 2016.1501 |
| dendritic cells | 2 | 89 | 531 | 102 | 2015.5882 |
| regulatory t-cells | 2 | 88 | 441 | 80 | 2015.2625 |
| immunity | 2 | 72 | 308 | 54 | 2015.6481 |
| tumor | 2 | 79 | 266 | 54 | 2015.6667 |
| melanoma | 2 | 78 | 278 | 51 | 2015.3725 |
| vaccine | 2 | 78 | 297 | 49 | 2015.2857 |
| her2 | 2 | 64 | 217 | 45 | 2016.2222 |
| antitumor immunity | 2 | 64 | 194 | 38 | 2015.1053 |
| responses | 2 | 68 | 211 | 38 | 2015.5526 |
| vaccines | 2 | 67 | 201 | 35 | 2014.8571 |
| antigen | 2 | 61 | 189 | 33 | 2015.5152 |
| metastatic breast | 2 | 59 | 215 | 33 | 2015.5455 |
| phase-i | 2 | 62 | 157 | 33 | 2016 |
| clinical-trial | 2 | 59 | 198 | 31 | 2016.2258 |
| monoclonal-antibody | 2 | 60 | 153 | 29 | 2014.3448 |
| gene | 2 | 49 | 121 | 26 | 2015.1154 |
| immune-response | 2 | 58 | 137 | 26 | 2015.3462 |
| group-study i-01 | 2 | 41 | 165 | 26 | 2015.4231 |
| peptide | 2 | 45 | 127 | 25 | 2014.64 |
| vaccination | 2 | 60 | 147 | 24 | 2014.5 |
| immune-responses | 2 | 54 | 130 | 24 | 2015.2083 |
| recurrence | 2 | 46 | 120 | 23 | 2016.3043 |
| her-2/neu | 2 | 54 | 136 | 22 | 2014.6818 |
| colony-stimulating factor | 2 | 46 | 118 | 21 | 2014.2857 |
| antibodies | 2 | 38 | 82 | 21 | 2015.1429 |
| risk | 2 | 46 | 92 | 21 | 2015.2857 |
| tumor-infiltrating lymphocytes | 3 | 87 | 956 | 170 | 2017.2 |
| survival | 3 | 85 | 505 | 98 | 2016.1837 |
| neoadjuvant chemotherapy | 3 | 69 | 379 | 65 | 2017.2154 |
| pd-l1 | 3 | 70 | 371 | 65 | 2018.0159 |
| triple-negative breast cancer | 3 | 78 | 298 | 58 | 2017.7241 |
| prognosis | 3 | 71 | 302 | 55 | 2016.2182 |
| antibody | 3 | 80 | 279 | 53 | 2015.9623 |
| pd-1 | 3 | 64 | 288 | 44 | 2017.186 |
| blockade | 3 | 73 | 246 | 44 | 2017.5 |
| poor-prognosis | 3 | 62 | 244 | 41 | 2017.1707 |
| pd-l1 expression | 3 | 58 | 243 | 38 | 2017.5 |
| prognostic value | 3 | 62 | 230 | 37 | 2017.2432 |
| triple negative breast cancer | 3 | 64 | 178 | 33 | 2017.1562 |
| safety | 3 | 56 | 183 | 30 | 2015.7667 |
| pathological complete response | 3 | 46 | 166 | 29 | 2017.1724 |
| estrogen-receptor | 3 | 53 | 140 | 28 | 2016.3214 |
| pembrolizumab | 3 | 51 | 162 | 28 | 2018.25 |
| subtypes | 3 | 55 | 142 | 26 | 2017.6538 |
| predictive-value | 3 | 52 | 164 | 25 | 2016.88 |
| antitumor-activity | 3 | 53 | 108 | 22 | 2017.1818 |
| nivolumab | 3 | 59 | 150 | 22 | 2017.8182 |
| association | 3 | 50 | 120 | 21 | 2016.7619 |
| chemotherapy | 4 | 96 | 726 | 139 | 2016.4604 |
| therapy | 4 | 89 | 626 | 129 | 2016.5349 |
| trastuzumab | 4 | 89 | 492 | 93 | 2016.3011 |
| combination | 4 | 74 | 210 | 44 | 2017.2093 |
| trial | 4 | 59 | 160 | 33 | 2016.6364 |
| phase-ii | 4 | 58 | 155 | 32 | 2016.9688 |
| tumors | 4 | 56 | 147 | 31 | 2016.5667 |
| efficacy | 4 | 52 | 154 | 28 | 2016.3214 |
| open-label | 4 | 36 | 112 | 28 | 2017.5357 |
| doxorubicin | 4 | 52 | 131 | 26 | 2016.6538 |
| double-blind | 4 | 47 | 98 | 24 | 2017.5417 |
| metastatic breast cancer | 4 | 50 | 92 | 23 | 2015.7391 |
| adjuvant chemotherapy | 4 | 55 | 115 | 22 | 2015.5455 |
| nanoparticles | 4 | 41 | 93 | 21 | 2017.65 |
| cyclophosphamide | 4 | 54 | 106 | 20 | 2014.85 |

Table S2. List of author keywords in the articles of topic 1 generated by VOSviewer.

| ID | Cluster | Link | Total Link Strength | Occurrence | Average publish year |
| --- | --- | --- | --- | --- | --- |
| monoclonal antibody | 3 | 1 | 1 | 5 | 2013.6 |
| angiogenesis | 2 | 3 | 3 | 7 | 2013.7143 |
| cancer-testis antigens | 4 | 1 | 1 | 5 | 2014 |
| cytokines | 1 | 7 | 7 | 6 | 2014.3333 |
| adcc | 4 | 7 | 7 | 5 | 2014.8 |
| ny-eso-1 | 4 | 6 | 7 | 5 | 2014.8 |
| breast neoplasms | 2 | 2 | 2 | 7 | 2015.1429 |
| metastatic breast cancer | 1 | 5 | 5 | 8 | 2015.25 |
| immunosuppression | 2 | 8 | 8 | 8 | 2015.25 |
| nk cells | 4 | 6 | 7 | 8 | 2015.25 |
| dendritic cells | 2 | 16 | 17 | 18 | 2015.3333 |
| vaccine | 4 | 4 | 5 | 6 | 2015.3333 |
| cytotoxicity | 1 | 6 | 6 | 5 | 2015.4 |
| apoptosis | 2 | 2 | 2 | 5 | 2015.4 |
| tumor microenvironment | 1 | 5 | 6 | 13 | 2015.5385 |
| immunity | 5 | 6 | 6 | 5 | 2015.6 |
| metastasis | 2 | 9 | 10 | 22 | 2015.8182 |
| chimeric antigen receptor | 6 | 5 | 6 | 8 | 2015.875 |
| her2/neu | 4 | 4 | 4 | 5 | 2016 |
| her2 | 1 | 8 | 12 | 17 | 2016.1765 |
| cancer immunotherapy | 6 | 5 | 5 | 10 | 2016.3 |
| regulatory t cells | 6 | 5 | 6 | 8 | 2016.375 |
| triple negative breast cancer | 6 | 5 | 5 | 13 | 2016.3846 |
| survival | 5 | 3 | 3 | 5 | 2016.4 |
| t cells | 4 | 6 | 7 | 6 | 2016.5 |
| dendritic cell | 1 | 2 | 2 | 5 | 2016.6 |
| immune response | 1 | 4 | 7 | 9 | 2016.6667 |
| macrophage | 2 | 4 | 5 | 6 | 2016.6667 |
| breast carcinoma | 5 | 5 | 10 | 6 | 2016.8333 |
| biomarker | 3 | 7 | 8 | 7 | 2016.8571 |
| immunogenic cell death | 1 | 4 | 5 | 5 | 2017 |
| inflammation | 2 | 5 | 5 | 6 | 2017 |
| prognosis | 3 | 14 | 22 | 27 | 2017 |
| cancer | 4 | 8 | 8 | 11 | 2017 |
| triple-negative breast cancer | 5 | 17 | 29 | 26 | 2017.1538 |
| immune checkpoints | 3 | 4 | 7 | 5 | 2017.2 |
| trastuzumab | 1 | 3 | 4 | 7 | 2017.2857 |
| pd-1 | 3 | 13 | 33 | 19 | 2017.3333 |
| natural killer cells | 5 | 3 | 3 | 11 | 2017.3636 |
| tumor-infiltrating lymphocytes | 3 | 7 | 10 | 8 | 2017.875 |
| pd-l1 | 3 | 15 | 47 | 36 | 2017.9143 |
| tnbc | 5 | 11 | 13 | 12 | 2017.9167 |
| immune checkpoint | 1 | 7 | 10 | 8 | 2018 |
| doxorubicin | 1 | 4 | 4 | 6 | 2018 |
| checkpoint blockade | 3 | 6 | 8 | 6 | 2018.3333 |

Table S3. List of author keywords in the articles of topic 2 generated by VOSviewer.

| label | cluster | Links | Total link strength | Occurrences | Average publish year |
| --- | --- | --- | --- | --- | --- |
| triple-negative breast cancer | 5 | 8 | 12 | 8 | 2018.875 |
| chemotherapy | 3 | 8 | 8 | 7 | 2017.1429 |
| pd-l1 | 2 | 7 | 9 | 5 | 2018.8 |
| trastuzumab | 4 | 3 | 3 | 4 | 2013.25 |
| metastatic breast cancer | 4 | 4 | 4 | 4 | 2015.25 |
| radiotherapy | 3 | 6 | 6 | 4 | 2016.25 |
| atezolizumab | 2 | 5 | 7 | 4 | 2018.75 |
| il-2 | 1 | 7 | 7 | 3 | 2015.3333 |
| carboplatin | 2 | 8 | 9 | 3 | 2016 |
| triple negative breast cancer | 5 | 1 | 1 | 3 | 2017.3333 |
| photothermal therapy | 1 | 5 | 5 | 3 | 2019 |
| high-dose chemotherapy | 4 | 5 | 5 | 2 | 2011.5 |
| muc1 | 6 | 1 | 1 | 2 | 2011.5 |
| dendritic cells | 4 | 2 | 2 | 2 | 2013.5 |
| glycated chitosan | 1 | 3 | 3 | 2 | 2014.5 |
| metastasis | 1 | 5 | 5 | 2 | 2015 |
| vaccine | 6 | 2 | 2 | 2 | 2015.5 |
| hyperthermia | 1 | 2 | 2 | 2 | 2016.5 |
| immune checkpoint blockade | 3 | 3 | 3 | 2 | 2017.5 |
| cancer immunotherapy | 3 | 3 | 3 | 2 | 2018 |
| checkpoint inhibition | 1 | 5 | 5 | 2 | 2018.5 |
| docetaxel | 2 | 1 | 1 | 2 | 2018.5 |
| triple negative breast cancer (tnbc) | 2 | 3 | 4 | 2 | 2018.5 |
| combination therapy | 5 | 3 | 3 | 2 | 2018.5 |
| immune checkpoint inhibitor | 6 | 2 | 2 | 2 | 2018.5 |
| overall survival | 2 | 4 | 5 | 2 | 2019 |
| immunochemotherapy | 5 | 2 | 3 | 2 | 2019 |
| immuno-oncology | 6 | 2 | 2 | 2 | 2019 |
